# Supplementary material for: Reading through the eyes of a university student: A double-masked randomised placebo-controlled cross-over protocol investigating coloured spectacle lens efficacy in adults with visual stress
Source: PLoS One. 2025 Jun 30;20(6):e0309625. doi: 10.1371/journal.pone.0309625 (PMC12208423; doi:10.1371/journal.pone.0309625)
Supplement: S2 File — (DOCX) [file pone.0309625.s002.docx]

**Reading Questionnaire**

**Personal Details:**

Name: _________________________________________ Gender (please circle): M / F / N

Age: _________________________________________

Email Address: ___________________________________________

Contact Number: __________________________________________

Undergraduate students are often required to do a lot of reading during their studies. Reading can be paper-based or screen-based. The following questionnaire relates to your vision when reading close-up print/text and whether you have any visual issues

1. Think about when you read. Do you ever experience any of the following symptoms when reading close-up print? If so, can you estimate on a scale of 0-5, how often you would experience these symptoms. 0 being ‘never’ and 5 ‘always’. Please insert a tick in the box that applies for each symptom. You can use the sample of print overleaf to help you answer these questions if you wish. Please ask the researcher if you have any questions or if there is something you do not understand

|  | **How often you have these symptoms when you read:** | | | | | |
| --- | --- | --- | --- | --- | --- | --- |
|  | Never | Rarely | Sometimes | Often | Most of the time | Always |
| **Symptoms** | 0 | 1 | 2 | 3 | 4 | 5 |
| Words/letters move |  |  |  |  |  |  |
| Words/letters merge |  |  |  |  |  |  |
| Words/letters look jumbled |  |  |  |  |  |  |
| Words/letters look blurred |  |  |  |  |  |  |
| Patterns/shadows seen in text e.g. rivers |  |  |  |  |  |  |
| Letters/words stand out in 3D above the page |  |  |  |  |  |  |
| Letters/words fade or darken on the page |  |  |  |  |  |  |
| Patterns seen in the white spaces between words/letters |  |  |  |  |  |  |
| Colours in the white spaces between words/letters |  |  |  |  |  |  |
| Headaches when reading |  |  |  |  |  |  |
| Eyestrain when reading |  |  |  |  |  |  |
| Repeating words/lines of text |  |  |  |  |  |  |
| Skipping words/lines of text |  |  |  |  |  |  |
| Losing your place when reading |  |  |  |  |  |  |
| Words/letters flicker when reading |  |  |  |  |  |  |
| Shimmering over the words/letters |  |  |  |  |  |  |
| Words/letters wobble when reading |  |  |  |  |  |  |

**Sample Text**

Det er ikke sa mye vi holder hellig i norskestammen var. Vi mangler felles grep om det sakrale. Lys, rekelse og inderlig religies kunst er ikke sertegn pa norsk kultur. Heller ikke samlet respekt for det andelige. Sett utenfra blir de felles ritualene vare oftest knyttet til det a ga tur. Ikke spasertur; men sliteturen. En sann en som barna blir dratt med pa fordi vi haper at de en gang vil forsta hvilken glede det gir for tanke og sjel at kroppen underveis blir skikkelig sliten. Da blir sendagen med den tradisjonelle turen en helligdag selv om forbindelsen med kirken svinger med felelsene. Sann var og typisk norske i dag. De er idrettsuteverne vi identifiserer det norske med. De som star opp mot smerte. Dess lenger pine, dess sterre helter. er det mulig a fele mer nasjonal glede gjennom idrett enn dengang de vant? De sterste norske navnene er de sterste sliterne er en gigantisk berg av og mot i verdens hardeste utholdenhetsidrett. Likevel vil han aldri bli hyllet av oss fra generasjon til generasjon. Det kan godt ga fort, men mest skal det ga langt. sakrale. Lys, rekelse og inderlig religies kunst er ikke sertegn pa norsk kultur. Heller ikke samlet respekt for det andelige. Sett utenfra blir de felles ritualene vare oftest knyttet til det a ga tur. Ikke spasertur; men sliteturen. En sann en som barna blir dratt med pa fordi vi haper at de en gang vil forsta hvilken glede det turen en helligdag selv om forbindelsen med kirken svinger med felelsene. Sann var og typisk norske i dag. De er idrettsuteverne vi identifiserer det norske med. De som star opp mot smerte. Dess lenger pine, dess sterre helter. er det mulig a fele mer nasjonal glede gjennom idrett enn dengang de vant De sterste norske navnene er de sterste sliterne er en gigantisk berg av og mot i verdens hardeste utholdenhetsidrett. Likevel vil han aldri bli hyllet av oss fra generasjon til generasjon. turen enhelligdag selv om forbindelsen med kirken svinger med felelsene. Sann var og typisk norske i dag. De er idrettsuteverne vi identifiserer det norske med. er en av de som har skjent det. Det er han som er den sterkeste forbindelsen mellom den gamle intervallstarten og seigpiningens nye kulisser.Underveis har han hatt sine kritiske meninger om hva som ble borte da langrenn gikk fra skog og starttid til stadion og fellesstart, men mest har han tilpasset seg konkurranseformen slik at det beste ble med videre.Det beste i pinesport er sannhets eyeblikk; akkurat da alle fysiske krefter egentlig er temt og du plutselig konkurrerer bare mot din egen brennende lyst til a gi opp. kilometer fer mal var den brannen inne i Estil tilsynelatende helt ute av kontroll. Da hadde han dratt feltet i stykker gjennom serpesneen i mil etter mil, men na var det han som var edelagt. Tetgruppa glapp; var der framme alene mot to tyskere og en tsjekker. var borte i sitt siste store internasjonale lep. med felelsene. Sann var og typisk og vi erI stedet ble Frodes kamp opp til teten igjen i den siste lange motbakken en sammenhengende jeg er fint, jeg har inge lyst a sove for tiden, men jeg skal ga til senge fort, men mest skal det ga langt. sakrale. Lys, rekelse og inderlig religies kunst er ikke sertegn pa norsk kultur. Heller ikke samlet respekt for det andelige. Sett utenfra blir de felles ritualene vare oftest knyttet til det a ga tur. Ikke spasertur; men sliteturen. En sann en som barna blir dratt med pa fordi vi haper at de en gang vil forsta hvilken glede det turen en helligdag selv om forbindelsen med kirken svinger med felelsene. Sann var og typisk norske i dag. De er idrettsuteverne vi identifiserer det norske med. De som star opp mot smerte. Dess lenger pine, dess sterre helter. er det mulig a fele mer nasjonal glede gjennom idrett enn dengang de vant De sterste norske navnene er de sterste sliterne er en gigantisk berg av og mot i verdens hardeste utholdenhetsidrett. Likevel vil han aldri bli hyllet av oss fra generasjon til generasjon. turen en helligdag selv om forbindelsen med kirken svinger med felelsene. Sann var og typisk norske i dag. De er idrettsuteverne vi identifiserer det norske med a fele mer nasjonal glede gjennom idrett enn dengang de vant De sterste norske navnene er de sterste sliterne er en gigantisk berg av og mot i verdens hardeste utholdenhetsidrett. Likevel vil han aldri bli hyllet av oss fra generasjon til generasjon. turen en helligdag selv

Rainbow Readers Ltd, 24 Westpark Cres, Falkirk, FK2 7GH, www.rainbowreaders.co.uk

©This is information has been compiled in association with RainbowReaders Ltd.

1. When looking at the sample text provided, do you experience any other symptoms not listed in the table on page 1? (please circle below)

Yes / No

If yes, can you please detail any other symptoms below:

__________________________________________________________________________________________________________________________________________________

1. Do you ever experience discomfort with certain artificial lights or light source flicker? (please circle below)

Yes / No

If yes, then;

- How often do you have this symptom?

________________________________________________________________

- When would you experience this symptom?

________________________________________________________________

1. Do you feel you have an aversion to striped patterns? (please circle below)

Yes / No

If yes, can you describe your aversion to striped patterns in more detail below please:

1. Have you had an eye test before? (please circle below)

Yes / No

If yes, when was your last eye test? ____________________________________________

1. Do you wear glasses / contact lenses (please circle below)

Yes / No

If yes, then continue to question 7

If no, then skip to question 10

1. Are you: short-sighted / long-sighted / have astigmatism / don’t know
2. Do you wear glasses / contact lenses: all the time / sometimes / occasionally / rarely

If you wear glasses / contact lenses part-time then continue to question 9.

If you wear glasses / contact lenses all the time skip to question 10.

1. If you don’t wear glasses / contact lenses all the time, what tasks do you wear them for? Please detail below:

_______________________________________________________________________

1. Have you ever had any of the following reading aids? (please circle as appropriate):

- Tinted lenses in glasses for reading: Yes / No
- Coloured piece of acetate paper to hold over near text when reading: Yes / No
- Coloured paper that reading material has been printed on: Yes / No
- Coloured filter applied to any electronic screens to assist reading: Yes / No

If yes to any of the above, then please give details below:

_________________________________________________________________________

1. Have you been diagnosed with any learning / reading difficulties? (please circle below)

Yes / No

If yes, then please give details below:

_________________________________________________________________________

1. Have you been diagnosed with dyslexia? (please circle below)

Yes / No

If yes:

- When were you diagnosed? _____________________________________________
- Who diagnosed the condition? ___________________________________________

1. Do any of your direct relatives (i.e. – Mother, Father, Siblings, Children) have dyslexia? (please circle below)

Yes / No

If yes, then which relative(s) have dyslexia?_______________________________________

1. Do you suffer regularly from headaches? (please circle below)

Yes / No

1. Do you suffer with migraine? (please circle below)

Yes / No

If yes:

- Have you been formally diagnosed with migraine? Yes / No
- How long have you had migraines for? ____________________________________
- How often would you have a migraine? ____________________________________
- How many migraines have you had in the last 3 months? ______________________

1. Do you have any diagnosed general health complaints? (please circle below)

Yes / No

If yes, please give details below:

_________________________________________________________________________

1. Do you take any medications? (please circle below)

Yes / No

If yes, please give details below:

_________________________________________________________________________

1. Do any of your direct relatives (i.e. – Mother, Father, Siblings, Children) have migraines?

Yes / No

If yes, then which relative(s) have migraines? _____________________________________

**Pattern Glare Test**

Using the string attached to ensure you are holding at 40cm from eye level.

Try to concentrate on the central square when looking at the pattern and consider the following questions. Please answer yes or no by circling the appropriate response


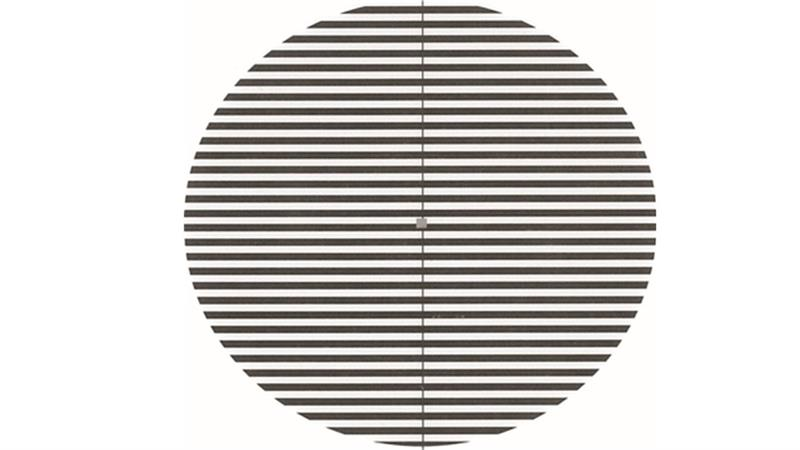


1. Do you see any colours within the stripes? Yes / No
2. Do you see any bending of the lines? Yes / No
3. Do you see any blurring of the lines? Yes / No
4. Do you see the lines shimmering or flickering? Yes / No
5. Do you see any of the lines fading or darkening? Yes / No
6. Do you see any shadowy shapes within the lines? Yes / No
7. Do you see any other effects while looking at the stripes? Yes / No

If yes, please give details below:

_________________________________________________________________________
