# Supplementary material for: Reading through the eyes of a university student: A double-masked randomised placebo-controlled cross-over protocol investigating coloured spectacle lens efficacy in adults with visual stress
Source: PLoS One. 2025 Jun 30;20(6):e0309625. doi: 10.1371/journal.pone.0309625 (PMC12208423; doi:10.1371/journal.pone.0309625)
Supplement: S3 File — (DOCX) [file pone.0309625.s003.docx]

**Protocol for Ethics Application.**

**Project Title**: Reading; through the eyes of a University student.

**Researchers**:

Dr. Sara McCullough

Chief Investigator

Lecturer in Optometry & Vision Science

School of Biomedical Sciences sj.mccullough@ulster.ac.uk

Tel: 70123078

Dr. Julie-Anne Little

Senior Lecturer in Optometry & Vision Science School of Biomedical Sciences

ja.little@ulster.ac.uk

Tel: 70124374

Mr Darragh Harkin

PhD Researcher, Optometrist

School of Biomedical Sciences

harkin-d13@ulster.ac.uk

**Background:**

Visual stress, also referred to as Meares-Irlen syndrome, Irlen syndrome or scotopic sensitivity, is a condition that can be characterised by symptoms of visual distortions which arise when looking at crowded, highly-contrasting, horizontal striped patterns such as text when reading. Some characteristic symptoms of visual stress include words moving, words merging, patterns through text, patterns in the white spacing between letters/words, letters standing out in 3D, letters fading or darkening, aversion to striped patterns, headaches when reading and sensitivity to glare and light flicker among others (Evans et al., 2017). The use of coloured filters (in the form of coloured overlays or lenses), have been reported to alleviate the symptoms of visual stress.

Historically it was believed that visual stress co-exists with dyslexia and many early studies recruited their subject base from a population of dyslexic children or adults (Gole et al., 1989; Menacker et al., 1993). However, with the development of different diagnostic criteria and methods of colour filter testing, visual stress is estimated to co-occur in only a small percentage (20%) of the population with dyslexia (Singleton and Trotter, 2005). Therefore, a large proportion of those with dyslexia do not have visual stress. It is now widely accepted that visual stress is an independent condition that can affect the general population independently of their dyslexia status.

Some literature has proposed that there may be a link between visual stress and other conditions including a family history of migraine (Maclachlan et al., 1993).

There are no established diagnostic criteria to identify visual stress. Diagnostic criteria vary across research papers, (Evans and Joseph, 2002; Wilkins, 2002a; Stein, 2014) even those written by the same authors (Wilkins et al., 1994; Bouldoukian et al., 2002; Evans and Joseph, 2002; Singleton and Trotter, 2005; Singleton and Henderson, 2007). Until recently, few attempts have been made to standardise the diagnostic criteria for visual stress; it is therefore not surprising that the prevalence of visual stress in the normal population is still debated. In a recent systematic review, Griffiths et al.

(2016) reports that the prevalence of visual stress across studies ranged from 13-96% of participants. This large range is unreflective of the population and is of little use in clinical optometric practice. Attempts have been made by Evans et al. (2017) through a Delphi study to help develop practical guidelines for the diagnosis of visual stress, however, these proposed guidelines have yet to be validated. Without validation and implementation of a robust diagnostic criterion of visual stress it is impossible to accurately determine the prevalence of visual stress in the general population.

The precise mechanism of visual stress remains unknown. However, it is hypothesised that it may be due to the over-stimulation of a hyperexcitable visual cortex caused by viewing high frequency horizontal striped patterns- i.e.- text (Wilkins et al., 1994). In theory, by altering the spectral composition of the retinal image (by viewing text through a coloured filter/lens), cortical activity can be altered, thus avoiding overexcitation in the visual cortex (Wilkins et al., 1984; Huang et al., 2003; Evans and Allen, 2016). Other literature has suggested that visual stress may be due to a deficit in the Magnocellular visual system. It is thought that the magnocellular pathway could play an important role in eye movements when reading due to it’s temporal processing function (Stein, 2001) If this is the case, then by improving Magnocellular function (by using coloured filters/lenses) reading performance could be improved (Malins, 2009). This theory receives criticism, partly due to the debate surrounding the precise colour required to aid visual stress, (Lightstone et al., 1999; Wilkins, 2002a; Stein, 2014) when the magnocellular system is not sensitive to colour processing.

Also, patients who have visual stress signs and symptoms do not consistently have an abnormal magnocellular visual system (Evans et al., 1995; Evans et al*.,* 1996; Simmers et al., 2001) and they do not consistently have coinciding difficulties with other tasks controlled by the magnocellular pathway (Simmers et al., 2001).

Regardless of hypothesis, it is proposed that the use of coloured filters alleviate the symptoms associated with visual stress. However, their use remains controversial. Despite many anecdotal claims and patient testimonies which supports the use of coloured filters, there is a lack of corroborating evidence available, (Griffiths et al., 2016; Evans and Allen, 2016) hence why they are not part of NHS eyecare practice. There are few robust studies that have systematically reviewed the effects of colour in adult participants (Evans and Allen, 2016; Griffiths et al., 2016). Griffiths et al. (2016) reports that the current available evidence assessing the effects of coloured filters on reading ability is of low quality. Those studies that have been conducted have been underpowered due to low participant numbers and/or ambiguous methodology and sampling criteria (Evans and Allen, 2016; Griffiths et al., 2016). The current available studies conducted to validate numerous colour filter systems have been affected by internal and external bias ranging from poor or no placebo control, participant selection and attrition (Griffiths et al., 2016).

There are different methods and systems available for prescribing coloured filters. One method is using a coloured overlay, this consists of a sheet of translucent coloured acetate which is placed over text, altering the spectral composition of the reading material. These overlays consist of different colours which are spaced around the CIE chromaticity diagram. Coloured overlays can be prescribed using different systems e.g.- Intuitive Overlays and Irlen Overlays. The other method uses coloured lenses determined by a range of different systems (Intuitive Colorimetry system, the Irlen system, ChromaGen/Harris lens system). The most widely used method in practice and research is Intuitive colorimetry^TM^ (Cerium Visual Technologies), which measures the chromaticity of precision tinted lenses.

Intuitive colorimetry^TM^ allows independent control of hue, saturation and brightness on a continuous basis. This allows rapid detection of a specific, precise tint to be chosen by the participant that alleviates visual stress symptoms. Current literature suggests that coloured lenses must be specifically prescribed to the subject in order to see the beneficial effects of colour in alleviating visual stress symptoms (Lightstone et al., 1999; Wilkins, 2002a). Thus, supporting the use of precision tinted lenses prescribed from the Intuitive Colorimeter as opposed to Intuitive overlays which are available in limited colours (nine colours spaced equally around the CIE diagram) (Lightstone et al., 1999).

It is not clear how an improvement in reading rate using standardised reading measures in the clinical setting, translates into real world improvements for the user. The most commonly used reading test for the investigation of visual stress is the Wilkins Rate of reading test (WRRT) which is often used as the only reading test in research studies (Wilkins et al., 1994; Wilkins, 2001; Singleton and Trotter, 2005). The WRRT is a highly standardised test of reading rate which consists of common, non-contextual words with narrow word spacing. Griffiths et al. (2016) criticise the use of the WRRT in isolation in visual stress research studies, favouring a test using more naturalistic text. Other attempts have been made to help ascertain the impact that coloured filters may have on a visual stress patients daily life. Wilkins et al. (1994) used a symptom diary to help identify the number of symptomatic versus symptom-free days in a population of children with visual stress who were given coloured spectacles for voluntary wear.

Visual stress is particularly relevant to the University student population. Students are often expected to read and comprehend large volumes of written material during their studies. With the advancement of portable electronics (smartphones, tablets, laptops) the average student can spend a significant proportion of their time reading text on an electronic screen. Glare and reflections are frequently identified as worsening the visual perceptual distortions associated with visual stress. Hence, text displayed on an electronic screen may exacerbate the visual stress symptoms perceived (Chu et al., 2011).

Considering current literature, the inconsistency in diagnostic criteria and poor quality of research evaluating the use of coloured filters to alleviate visual stress, it is clear that visual stress could go undetected and undiagnosed in the population. There could be a significant proportion of the student population who could have undiagnosed visual stress and could benefit from treatment to aid reading ability in their studies.

**Aim:**

The aim of this research project is to investigate the prevalence of visual stress in the adult student population and to assess if there are any co-existing factors (history of migraine, dyslexia) simultaneously evaluating the diagnostic criteria to help identify and manage visual stress. The project aims to evaluate the effect of coloured lenses on visual stress clinical signs and symptoms and to compare visual stress symptoms on an electronic screen compared to paper-based text.

**The specific aims of this research study are:**

**Part 1:**

1. To estimate the proportion of visual stress in the adult student population.
2. To determine how many adult students who have visual stress symptoms may have coexisting conditions including dyslexia and migraines.

A subset of participants who have been identified through Part 1 of this study as potentially having visual stress will be invited to participate in Part 2 of this study to address the following specific objectives:

**Part 2:**

1. To validate the visual stress diagnostic criteria proposed in a recent Delphi study (Evans et al., 2017) by utilising a reading symptoms questionnaire and pattern glare test.
2. To assess the effect of coloured filters on visual stress symptoms, rate of reading and pattern glare test results.
3. To compare how visual stress symptoms manifest on a screen-based source when compared to paper-based text.
4. To assess if improvements in reading rate can have real-world improvements in reading ability.

**Methods:**

Part 1 of this study will be conducted on 580 participants. This has been calculated using the powerbased sample size calculation below in order to obtain a sufficient number of participants for Part 2 of this study.

𝟐 ^𝟐^ 𝟐(𝒁𝟏−𝜷+𝒁𝟏−𝜶/𝟐) 𝝈𝒘

𝒏 = 𝒅_𝟐_

where 𝒏 is the total sample size, 𝝈_𝒘_ is the within-subject standard deviation, 𝒅 is the expected difference between the two treatments and 𝒁_𝟏−𝜷_ and 𝒁_𝟏−_𝜶 are the normal values for power and

𝟐

significance.

The Wilkins Rate of Reading Test reading speed has been used as the outcome measure for an improvement with the coloured lenses. A within-subject standard deviation of 10.85 words per minute has been calculated from estimated values from repeat measures data on a sample of primary school children within Wilkins et al. (1996). The expected differences between the two treatments has been estimated as 6.5 words per minute derived from data on a non-dyslexic population of adults with visual stress in Singleton and Trotter (2005). A power of 90% and a significance level of 0.05 are set as the normal values. A sample size of 58.7 ≈ **59** participants is required. This has been increased by 10% to **65** to account for attrition. To obtain these numbers via the screening study, given that approximately 75% of those suitable may participate, a screening sample study size of **580** is required using a conservative prevalence visual stress of 15% within an unselected population based on data from Singleton and Henderson (2007).

580 participants will be recruited from the Ulster University student population. The research team will contact the Course Directors of large programmes on the Coleraine campus, and, with their agreement will liaise with module coordinators for Year 1 and Year 2 modules.

Recruitment will be conducted as follows:

1. Module coordinators will be asked to circulate information about the study to the students via email or Blackboard announcements. The study information circulated includes a short summary of the study (Appendix 1) and a participant information sheet (PIS Part 1).
2. This will be followed up with the researcher attending a lecture where time has been allocated for the researcher to address the class cohort. The researcher will explain the exclusion and inclusion criteria to the group in order to reduce inappropriate participation.
3. Students who then wish to participate will be asked to remain in the lecture theatre and will be asked to sign a consent form (Appendix 2).

Exclusion criteria includes anyone who has or has had a history of photosensitive epilepsy due to the use of the pattern glare test which carries a small risk of seizure in those with this condition. This is on the PIS Part 1 and will also be reiterated by the researcher when addressing the class, prior to the participants signing the consent form.

Those with a diagnosis of dyslexia or migraine will not be excluded from participation but will be noted for analysis purposes on the data collection sheet.

**Protocol and Techniques:**

Data collection for Part 1 of the study will be conducted within lecture theatres on the Coleraine Campus, Ulster University. All data collection for Part 2 of the study will be performed in the Optometry clinic on the Coleraine campus, Ulster University.

Part 1 and Part 2 of this study are masked studies, hence the participant will deliberately not be given information about the full aims of this study, instead they will be advised that this is a study about reading difficulties (evident in the Participant information sheets, PIS Part 1 and PIS Part 2). This is to minimise bias throughout the study. The participant will be kept fully informed about all the tests and procedures conducted, none of which are invasive or will directly or indirectly cause harm to the participant. The participant will be given minimal information about the ‘experimental’ colour and ‘control’ colour used for the tinted lenses in the participant information sheet Part 2 and throughout Part 2 of the study.

Appendix 3 illustrates a flowchart visually representing the protocol of Part 1 and Part 2 of this study.

**Part 1:**

- The Reading Questionnaire and Pattern glare test are available in Appendix 4. The questions on the questionnaire have been adapted from the Delphi study (Evans et al., 2017). The sample of text used in the questionnaire has been developed by Rainbow Readers Ltd, it is routinely used in the visual stress clinical setting as a questionnaire tool. This section of the study is brief, lasting approximately 10 minutes per participant and will be completed individually by each participant in their class groups (n~100 students per group).

**Part 2:**

- The participants identified with potential visual stress symptoms via Part 1 of this study will be contacted (provided follow-up consent has been given). They will be sent the Participant information sheet, Part 2 (PIS Part 2) and will be asked to sign a consent form for inclusion in Part 2 of this study (Appendix 5).
- Basic Optometric eye tests will be performed to help screen participants to ensure they meet the inclusion criteria for Part 2 of this study. These tests are regularly used in routine clinical eye care and will be used to help identify any focussing difficulties (accommodative insufficiency), eye muscle issues (convergence insufficiency), binocular vision problems (heterotropia or decompensating heterophoria), reduced vision and/or significant uncorrected spectacle prescription. All these tests will be performed by a GOC-registered Optometrist. None of these tests are invasive or touch the eye. They don’t involve any bright lights. The participant can take breaks where needed. These tests will take approximately 25 minutes per participant and will be conducted on approximately 80-100 participants.
- The visual stress tests will be conducted on those participants who meet the inclusion criteria for Part 2 of this study (n=65). These tests should take approximately 25 minutes per participant to complete. These tests include:
  1. Wilkins Rate of reading test, which is a test measuring the rate of reading. The participant is asked to read aloud the words as quickly and accurately as they can in one minute (Wilkins et al., 1996).
  2. Pattern glare test (same as in Part 1 of this study, in Appendix 4). The participant is asked to look at a striped pattern and to comment on what they see, e.g. colours, stripes moving/bending etc. (Evans et al., 2017)
  3. A Naturalistic text reading assessment. This will consist of a passage of text and the participant will be asked to read the words as quickly and accurately as possible in one minute.
  4. Colorimetry assessment using the Intuitive Colorimeter^TM^. (The full procedure is described in detail in Wilkins [2002b]) In brief, the participant will be asked to look into a box with random text. The examiner will then present colours across the full colour spectrum and the participant will be asked which colour is the optimum to alleviate their reading difficulty symptoms compared to reading text with no colour.

This does not involve any bright lights and nothing will touch the participant’s eyes. The participant can take breaks where needed.

- - 1. The ‘**experimental’ colour** will be determined as the optimum chromaticity to help alleviate visual stress symptoms using the methodology described by Wilkins, (2002b). This colour will be used as the colour of tint for the ‘experimental’ coloured lenses.
    2. The ‘**control’ colour** will be determined for each participant. This colour will have a chromaticity which is not aversive to the wearer and does not exacerbate symptoms of visual stress. This colour will be used as the colour of tint for the ‘control’ coloured lenses.

1. There will be other tests conducted on the participants first visit. These are routine non-invasive tests which are performed regularly in the visual stress clinic. These tests include:
   - Colour vision assessment using the City colour vision test.
   - The Developmental Eye Movement test (DEM): this test assesses how long it takes the participant to read aloud numbers with irregular horizontal spacing.
   - The Test of Word Reading Efficiency (TOWRE): this assesses the participants rate of reading nonsense words (using their phonic skills) compared to reading common words.
   - Eye movements test: the participant is asked to carefully fixate their gaze on a swinging pendulum, their eye movements are graded by the examiner using an eye tracking device.
   - Eye dominance: this test assesses if the participant has a dominant eye (their preferred eye they use to see) and which eye this may be. Nothing will touch the participants eye and there are no bright lights involved. The participant can take breaks where needed. The results of these tests will be used when analysing the data collected. These tests will take approx. 15 minutes (n=65).
2. The screen-based tests will be the same as used above in 1-3 viewed on a tablet screen instead of paper-based. These will be performed without any coloured spectacles on the first spectacle collection visit. This will take approximately 15 minutes (n=65).
3. The participant will be given each set of coloured lenses in a spectacle frame in a randomised order. 50% of participants will receive the ‘experimental’ coloured spectacles first, followed by a ‘wash-out’ period which is a minimum of six weeks of no coloured spectacle wear, followed by the ‘control’ coloured spectacles and vice versa. The participant will be given each pair of spectacles for a minimum of 10 weeks to wear. They will be advised that the spectacles are recommended to be worn for concentrated tasks with the exclusion of night-time driving. The participant will be aware they can wear the spectacles as and when they want to. At the initial collection of the spectacles and when the participant returns the spectacles the WRRT, natural text and pattern glare test will be repeated with the coloured spectacles in place.
4. The participant will be asked to keep a simple symptom diary (Appendix 6). They will be instructed on how to complete this accurately. The symptom diary will be sent to the participant via a link to a survey. The symptom diary will be completed daily for the first week and final week of wear of both set of coloured spectacles.
5. After both the ‘control’ and ‘experimental’ coloured spectacles have been worn by the participant, the participant will be given their preferred set of coloured lenses in the spectacle frames to keep. They can wear them as and when they want to.
6. Approximately 4 months following the end of this study the participant will be contacted via telephone (provided follow-up consent has been given) to see if they are still wearing the coloured spectacles voluntarily.

**Data protection issues:**

Data will be treated and stored in line with the Data Protection Act. The reading questionnaire will have personal information attached so as to allow the examiner to identify those participants with the relevant symptoms so as they can be contacted to participate in the next section of the study. Once collected and analysed the questionnaire sheets will be anonymised by assigning each participant a unique participant number. The completed questionnaire will be held in a secure office in a locked filing cabinet which is only accessible by the involved research team The participants in the second section of this study will be identified using their participant number from Part 1 of the study, so as their data collection sheets can remain anonymous. The information that connects the participant to their unique number will be stored on a password encrypted desktop kept in a secure room which can only be accessed by the involved research team.

**Statistical Analysis:**

The proportion of visual stress in the sample student population will be determined as the number of individuals classified as having visual stress divided by the total number of participants (%). The proportion of those who co-exhibit dyslexia and migraine will be examined.

Descriptive statistics will be used initially to explore the results. The percentage of days where spectacles were worn and duration of wear will be compared between the ‘experimental’ and ‘control’ spectacles. McNemar tests of proportions and paired t-tests will be used to analyse statistical differences between frequency and duration of wear of the ‘experimental’ and ‘control’ spectacles.

Descriptive statistics for reading speed and ability prior to using the coloured lenses, with the ‘experimental’ lenses and with the ‘control’ lenses will be explored. Improvements in reading will be analysed using repeat measures ANOVA. The proportion of symptom free days versus days with symptoms out of the number of days the spectacles were worn will be compared between the ‘experimental’ and ‘control’ arms.

The number and severity of visual stress symptoms reported using paper-based text and pattern glare test versus that displayed on an electronic screen will be compared using Chi-squared analysis.

The visual stress diagnostic criterion proposed following the Delphi study by Evans et al. (2017) will be evaluated. This will be evaluated by comparing the symptoms (identified through the reading questionnaire), the PGT results, WRRT and reading tests of those who voluntarily continue to use the experimental coloured spectacles for 4 months to those who did not continue with voluntary wear or who continued with wear of the control spectacles.

**Ethical Considerations:**

All aspects of this study are low risk and non-invasive. All the testing methods used are non-invasive, low risk procedures which will be conducted by a GOC-registered Optometrist in the Optometry Clinic. The research team are familiar with all the relevant Health and Safety policies and procedures in place.

Participants who have or have had a history of photosensitive epilepsy are excluded from participating in any element of the study due to the use of patterns and crowded text that could potentially trigger a seizure in those with this condition.

When the participants are given each pair of spectacles to wear, they will be advised to wear the spectacles for concentrated tasks, excluding driving at night due to the reduced transmission of the coloured lenses. Participants will be advised that they can wear the spectacles as and when they want to.

This study could identify a range of Optometric issues ranging from reduced vision, uncorrected glasses prescription, undetected ocular muscle weakness or accommodation weakness. If any of these issues are detected then, with consent, the participant will be given the option of attending an Optometric appointment in the Optometry Clinic at Ulster University (Coleraine) or they will be directed to their own community Optometrist.

This study could identify undiagnosed visual stress. If visual stress is identified throughout this study, the relevant vision tests will be conducted as part of this study. If there are any participants with symptoms suggestive of visual stress that cannot participate in this study for any reason, they will be signposted to the Visual Stress clinic in the Optometry Clinic, Coleraine, Ulster University. Visual stress can co-exist with some learning and reading difficulties. If it was suspected that the participant may have reading difficulties or another difficulty then the participant will be signposted to their course director/tutor and can be referred to student support services within the University who can arrange for further help, guidance and support.

**Dissemination of Research Results:**

The results of this project will be disseminated to researchers and health professionals through presentations at conferences and publications in peer-reviewed articles.

**References:**

Bouldoukian, J., Wilkins, A. and Evans, B. (2002) Randomised controlled trial of the effect of coloured

overlays on the rate of reading of people with specific learning difficulties. Ophthalmic and Physiological Optics, 22(1), 55–60.

Chu, C., Rosenfield, M., Portello, J., Benzoni, J. and Collier, J. (2011) A comparison of symptoms after viewing text on a computer screen and hardcopy. Ophthalmic Physiological Optics, 31(1), 29-32.

Evans, B. and Joseph, F. (2002) The effect of coloured filters on the rate of reading in an adult student population. Ophthalmic and Physiological Optics, 22(6), 535–545.

Evans, B., Allen, P. and Wilkins, A. (2017) A Delphi study to develop practical diagnostic guidelines for visual stress (pattern-related visual stress). Journal of Optometry, 10(3), 161-168.

Evans, B. and Allen, P. (2016) A systematic review of controlled trials on visual stress using Intuitive Overlays or the Intuitive Colorimeter. Journal of Optometry, 9(4), 205-218.

Gole, G., Dibden, S., Pearson, C., Pidgeon, K., Mann, J., Rice, D., Rooney, K., Hannell, G., Fitzgerald, B. and Kortman, J. (1989) Tinted lenses and dyslexics–a controlled study. Australian and New Zealand Journal of Ophthalmology, 17(2), 137–141.

Griffiths, P., Taylor, R., Henderson, L. and Barrett, B. (2016) The effect of coloured overlays and lenses on reading: a systematic review of the literature. Ophthalmic and Physiological Optics, 36(5), 519-544.

Henderson, L., Tsogka, N. and Snowling, M. (2013) Questioning the benefits that coloured overlays can have for reading in students with and without dyslexia. Journal of Research in Special Educational Needs, 13(1), 57–65.

Huang, J., Cooper, T., Satana, D., Kaufman, D. and Cao, Y. (2003) Visual distortion provoked by a stimulus in migraine associated with hyperneuronal activity. Headache, 43(6), 664–671.

Jeanes, R., Busby, A., Martin, J., Lewis, E., Stevenson, N., Pointon, D. and Wilkins, A. (1997) Prolonged use of coloured overlays for classroom reading. British Journal of Psychology, 88, 541–548.

Lightstone, A., Lightstone, T. and Wilkins, A. (1999) Both coloured overlays and coloured lenses can improve reading fluency, but their optimal chromaticities differ. Ophthalmic and Physiological Optics 19(4), 279–285.

Maclachlan, A., Yale, S. and Wilkins, A. (1993) Open trials of precision ophthalmic tinting: A follow‐up of 55 patients. Ophthalmic and Physiological Optics, 13(2), 175–178.

Malins, C. (2009) The use of coloured filters and lenses in the management of children with reading difficulties. A literature review commissioned for the Irlen Screening research project. Mind Matters Psychology. pp 18-20.

Menacker, S., Breton, M., Breton, M., Radcliffe, J. and Gole, G. (1993) Do tinted lenses improve the reading performance of dyslexic children? A cohort study. JAMA Ophthalmology, 111(2), 213–218.

Mitchell, C., Mansfield, D. and Rautenbach, S. (2008) Coloured filters and reading accuracy, comprehension and rate: a placebo-controlled study. Perception Motor Skills Journal 106(2), 517– 532.

Simmers, A., Bex, P., Smith, F. and, Wilkins A. (2001) Spatiotemporal visual function in tinted lens wearers. Investigative Ophthalmology and Visual Science, 42(3), 879–884.

Singleton, C. and Trotter, S. (2005) Visual stress in adults with and without dyslexia. Journal of Research in Reading, 28(3), 365–378.

Singleton, C. and Henderson, L. (2007) Computerized screening for visual stress in children with dyslexia. Dyslexia (Chichester England), 13(2), 130–151.

Stein, J. and Walsh, V. (1997) To see but not to read; the magnocellular theory of dyslexia. Trends in Neuroscience, 20(4), 147-152.

Stein, J. (2001) The Magnocellular Theory of Developmental Dyslexia. Dyslexia, 7(1), 12-36.

Stein, J. (2014) Dyslexia: the role of vision and visual attention. Current Development Disorders Report, 1(4), 267–280.

Wilkins, A., Nimmo-Smith. I., Tait, A., McManus, C., Sala, S., Tilley, A., Arnold, K., Barrie, M. and Scott, S. (1984) A neurological basis for visual discomfort. Brain, 107, 989-1017.

Wilkins, A., Nimmo-Smith, I. and Jansons, J. (1992) Colorimeter for the intuitive manipulation of hue and saturation and its role in the study of perceptual distortion. Ophthalmic Physiological Optics, 12(3) 381-385.

Wilkins, A., Evans, B., Brown, J., Busby, A., Wingfield, A., Jeanes, R. and Bald, J. (1994) Doublemasked placebo-controlled trial of precision spectral filters in children who use coloured overlays. Ophthalmic Physiological Optics, 14(4), 365–370.

Wilkins, A., Jeanes, R., Pumfrey, P. and Laskier, M. (1996) Rate of Reading Test®: its reliability, and its validity in the assessment of the effects of coloured overlays. Ophthalmic Physiological Optics, 16(6), 491-497.

Wilkins, A., Lewis, E., Smith, F., Rowland, E. and Tweedie, W. (2001) Coloured overlays and their benefit for reading. Journal if Research in Reading, 24(1), 41–64.

Wilkins, A. (2002a) Coloured overlays and their effects on reading speed: a review. Ophthalmic Physiological Optics, 22(5), 448–454.

Wilkins, A, (2002b) A System for Precision Ophthalmic Tinting. Manual for the Intuitive Colorimeter Mk.2 and precision tints. Visual Perception Unit, University of Essex, Colchester, UK, Cerium Visual Technologies, Tenterden, Kent, UK. 6^th^ edition. Accessed online: <https://www1.essex.ac.uk/psychology/overlays/mk2ukman.pdf>

Wilkins, A., Allen, P., Monger, L. and Gilchrist, J. (2016) Visual stress and dyslexia for the practicing optometrist. Optometry in Practice, 17, 103–112.
