# Supplementary material for: Reading through the eyes of a university student: A double-masked randomised placebo-controlled cross-over protocol investigating coloured spectacle lens efficacy in adults with visual stress
Source: PLoS One. 2025 Jun 30;20(6):e0309625. doi: 10.1371/journal.pone.0309625 (PMC12208423; doi:10.1371/journal.pone.0309625)
Supplement: Attachment — Submitted filename: Response to Reviewers. .docx [file pone.0309625.s005.docx]

**Response to Reviewers**

**Manuscript Title:** Reading through the eyes of a university student: A double-masked randomised placebo-controlled cross-over protocol investigating coloured spectacle lens efficacy in adults with visual stress.

**PLOSOne Reference:** PONE-D-24-33918

Dear Academic Editor & Peer reviewers,

Thank you for your insightful and thorough comments, which we have used to strengthen and develop our manuscript: “Reading through the eyes of a university student: A double-masked randomised placebo-controlled cross-over protocol investigating coloured spectacle lens efficacy in adults with visual stress.”, for consideration of publication in *PLOSOne*. An edited document with highlighted changes to the text has been included in the re-submission. To improve readability, addressed comments from reviewer #1 are highlighted in purple text, with amendments made from reviewer #2’s comments are noted in red text. Where both reviewers raised the same point and an amendment was made, altered text has been written in green text. Detailed point-by-point response to each of the peer reviewers’ queries can be seen below, with reference to which line number(s) in the manuscript, which have been amended are also included.

**Reviewer’s Comments to Authors**

Reviewer 1:

**The protocol is a longitudinal study of colored lenses in reading for university students. There are numerous statistical deficiencies due to the terseness of the protocol:**

**Author Response:** Thank you for your comment in identifying potential statistical issues. We appreciate your comment and have amended the section on statistical analysis in lines 410 to 437.

**1. The randomization procedure is not specified (permuted blocks, complete randomization, e.g.).**

**Author Response**: Thank you for identifying this omission. We have now amended the manuscript on lines 275 to 278, to inform the reader that the randomisation procedure is a 1:1 randomisation without stratification. Further, we provide a rationale as to why this methodology was employed in the amended manuscript.

**There is no discussion of the prevention of ascertainment or selection bias.**

**Author Response:** Thank you for mentioning a potential source of bias that may develop during our trial. We have added a paragraph to our discussion, identifying the limitations of this trial. We have acknowledged the potential for ascertainment and selection bias and have discussed methods which we will employ to mitigate these issues. The discussion can be found on lines 485 to 501.

**2. The sample size is based on a treatment difference seen in prior studies. Studies are powered for a CLINICALLY MEANINGFUL DIFFERENCE, not the observed effect size for prior studies.
3. No details are given on what sample size computations are used; since the outcome is based on a repeated measures ANOVA, there need to be measures of variability and covariance in order to compute the sample size. No indication is given as to the distribution of the primary outcome, which appears to be a count or a score based on words.**

**Author Response:** Thank you for raising these points in relation to sample size calculations. We acknowledge that our initial sample size calculation may have been erroneous in nature and have re-calculated our data for a repeated measures ANOVA as our primary outcome measure. Using statistical software (G-Power, Version 3.19.4, 2024), we have calculated an a priori sample size for a within-factors repeated measures ANOVA. A partial eta squared (${\eta\rho}^{2}$) of 0.0355 was used with a conservative small to medium effect size ($f$) of 0.1985. Probability of a type-1 error ($\alpha$), of 0.05 and power ($1-\beta$) of 0.90 remained unchanged. As this is a crossover trial, only one analysable group are to be considered with 3-repeat measures of reading speed: at baseline, upon initial spectacle collection and after 6-weeks of spectacle wear. Correlation among repeat measures was assumed 0.5 and nonsphericity correction ($\epsilon$) was held at 1.0. This calculation generated a sample size of n=59. These details have been included in lines 124 to 129 of the manuscript.

**4. There are no details on the statistical modelling, what model will be used, if any covariates will be examined, what model assessment diagnostics will be used, what modelling assumptions will be made.**

**Author Response:** Thank you for this consideration. We appreciate your suggestion to assess for covariates. Statistical differences in our primary outcome measures will be determined by repeated measures ANOVAs. However, we will consider the effects of covariates such as baseline reading speed, hours spent wearing spectacle and number of days wearing spectacles in post-hoc analysis through repeated measures ANCOVA testing, as required. This comment is addressed in lines 415 to 418. As this is a relatively homogenous population of undergraduate university students of similar age, other covariates are not considered relevant.

**5. "Repeat" measures ANOVA should be "Repeated" measures ANOVA.**

**Author Response:** Thank you for pointing out this mistake. We have amended the protocol and have changed the word ‘repeat’ to ‘repeated’ on line numbers 411, 414 and 429.

**6. I don't understand the first part of the title, and the punctuation after "Reading".**

**Author Response:** We thank the reviewer for this comment and apologise for making it difficult to understand. The first part of the title is used in all participant documentation within the study to allow a suitably vague title to prevent participants having prior knowledge of the protocol to avoid potential study bias. This title is also linked to the study protocol held on the Clinical Trials website (<https://clinicaltrials.gov/study/NCT04318106?cond=visual%20stress&rank=7>). We appreciate the first part of the title does not help inform the reader of the content of the publication but would be grateful it if could remain to link the Clinical Trials registration with the current protocol publication. We appreciate that the use of the semi-colon may be redundant. As such, we have removed this from the trial name. This change can be seen in line 4.

Reviewer 2:

**Thank you for a well written protocol that helpfully covers most areas of the SPIRIT guidelines. I have several comments that you should consider to improve the manuscript.**

**Author Response:** Thank you for your comprehensive and constructive comments. We have found this feedback highly beneficial at improving the strength of our manuscript. Individual changes to the manuscript are detailed below.  **Introduction
The three paragraphs are do not follow a logical flow for me. I suggest: visual stress including prevalence and significance, aetiology and evidence supporting proposed mechanisms, treatment options including evidence for and against, professional guidelines, primary aim of trial.**

**Author Response:** Thank you for your feedback. The introductory paragraphs have been re-drafted to follow the three areas suggested. The amended paragraphs occupy lines 64 to 102 of the manuscript. **You state “in small scale RCTs” and yet any RCT should be powered to find a difference i.e. scale does not matter. I suggest your statement is unpacked further. For example, do all trials show reduced symptoms and is this statistically and/or clinically significant?**

**Author Response:** Thank you for highlighting this. We accept that stating “small scale RCTs” may not be appropriate and have re-worded this. We have now addressed both statistically and clinically significant differences in reading speeds in previous randomised control trials and how these vary greatly in a selection of previous studies reported in lines 92-96. **Study Design
What is your argument for a two-week washout period?**

**Author Response:** We have now described the rationale for a 2-week washout period in the manuscript, included in lines 306 to 308. Two reasons are outlined. Firstly, this time period is equivalent to previous randomised control trials of precision tinted lenses, investigating precision tinted lens efficacy. Secondly, it is proposed that this is an appropriate interval to which colour memory for a chosen colour is negligible – as described further by D’Ath et al (2007).

References:

D’Ath P, Thomson W, Wilkins AJ. Memory for the color of non-monochromatic lights. *Col. Res. Appl.* 2007; 32: 11-15 DOI:  <https://doi.org/10.1002/col.20281>

**One concern here is that there must be some uncertainty in determining a tint that lies just outside the Limits of Therapeutic Effect but also appears similar enough for masking. It would be good to argue this clearly given some (limited) evidence from Suttle et al (2017) that results are variable.**

**Author Response:** Thank you for this insightful comment. We acknowledge that there is a certain degree of variability in how we will ensure that the tint lies outside the Limits of Therapeutic Effect. We have expanded our methodology in lines 260 to 265, stating that the researchers will attempt to ensure that the control tint remains ‘inert’ through confirmation of participants’ visuoperceptual reading symptoms when viewing text under the identified control chromaticity. We appreciate that the work of Suttle et al (2017) suggests variability in results. One way in which the authors propose that variability in results may be minimised, is that all baseline data measures (including) Intuitive Colorimetry, will be conducted by a single examiner D.H., reducing any potential risk of interpractitioner variability, as discussed further in lines 324 to 327.

References:

Suttle CM, Barbur J, Conway ML. Coloured overlays and precision-tinted lenses: poor repeatability in a sample of adults and children diagnosed with visual stress. *Ophthalmic Physiol. Opt.* 2017; 37: 542-548 DOI: <https://doi.org/10.1111/opo.12389>
 **Study Population
Good to argue why you have chosen a student population because it would seem difficult to extrapolate to a wider population affecting the generalisability of your findings. For example, it is reasonable to anticipate an age range clustered around 18-24 with less participants as you go to your upper age limit of 45 (unless you are using stratified sampling). Also, the amount of reading your sample does will be higher than the general population.**

**Author Response:** Thank you for this observation. We are in agreement that a student population may not be wholly representative of the wider adult population. Yet, researchers are confident that setting upper age-limit of 45-years, will avoid recruitment of participants experiencing reading discomfort as a result of age-related uncorrected refractive error – i.e. presbyopia. This is discussed further in lines 136 to 140. As this investigation is a superiority trial, our primary outcome measure is to determine if a clinically significant benefit can be endorsed with use of precision tinted lenses. Furthermore, this trial will explore coloured lens efficacy in a cohort of subjects with an equitable baseline level of reading competence, as all undergraduate students enrolled will have to exhibit demonstrable English language competence to enrol at Ulster University. If benefit of coloured lenses can be determined through this study, the authors appreciate that repeat investigations will be needed in generic/stratified populations, which may more accurately represent the adult population in the U.K or indeed a paediatric population. We reference these study limitations in lines 485 to 501.  **Sample size Calculation
Did you assume a RM ANOVA when carrying out this power calculation? At a basic level the SD of the difference is fundamentally important rather than the within subjects SD, which could differ between groups. Could this be given?**

**Author Response:** Thank you for this useful comment. As also addressed by reviewer 1, we have now adjusted our sample size calculation for repeated-measures ANOVA. We have re-calculated our data for a repeated measures ANOVA as our primary outcome measure. Using statistical software (G-Power, Version 3.19.4, 2024), we have calculated an a priori sample size for a within-factors repeated measures ANOVA. A partial eta squared (${\eta\rho}^{2}$) of 0.0355 was used with a conservative small to medium effect size ($f$) of 0.1985. Probability of a type-1 error ($\alpha$), of 0.05 and power ($1-\beta$) of 0.90 remained unchanged. As this is a crossover trial, only one analysable group are to be considered with 3-repeat measures of reading speed: at baseline; upon initial spectacle collection and after 6-weeks of spectacle wear. Correlation among repeat measures was assumed 0.5 and nonsphericity correction ($\epsilon$) was held at 1.0. This calculation generated a sample size of n=59. These details have been included in lines 124 to 129 of the manuscript.

**Recruitment
This section has sub-sections on screening tests and doesn’t comment on recruitment of participants!**

**Author Response:** Thank you for this comment. We have now amended the manuscript in lines 219 to 222 to explain the recruitment process. **Outcome Measures
Please ensure the exact metric for the outcome measure is reported. And also indicate if it is an average or median measure for example and if any repeats are used.**

**Author Response:** Thank you for your feedback. Average repeated measures ANOVA with and without coloured lenses will be conducted for the following reading tests, when assessing reading speed: Wilkins Rate of Reading Test; Adult Reading Test (2^nd^ Edition); Test of Word Reading Efficiency; Developmental Eye Movement Test. As the pattern glare test can only be scored on an ordinal scale (i.e. possible pattern glare scores are limited to values of 0,1,2,3,4,5,6 and 7), median change in pattern glare test score will be reported. A test of normality (Shapiro-Wilk Test), will be conducted when conducting statistical analysis. In the event that data is non-normally distributed, non-parametric equivalents of the statistical tests employed will be assessed; namely a Friedman test in place of repeated measures ANOVA. As McNemar’s Test of Proportions is non-parametric in nature, this will not need to be adjusted. These amendments are included in lines 343 to 344, 410 to 415 and 433 to 434.

**Why are you using a percentage reading speed change? Percentages are not normalised and relate to different effect size changes.**

**Author Response:** We appreciate that percentages are not-normalised in nature. We will be reporting percentage change alongside whether there is a statistically significant change in Wilkins Rate of Reading Test Reading Speed by repeated measures ANOVA. Percentage change in reading speed has frequently been used to measure clinically-significant improvement in reading performance with/without coloured lenses and in recent investigation, has been proposed indicative and potentially diagnostic of visual stress – see Evans et al (2017). We accept the reviewer’s comment on the non-normalised nature of percentages and as such will amend our statistical plan to additionally report effect sizes. This amendment can be seen on lines 348 to 353.

References:

Evans BJW, Allen PM, Wilkins AJ. A Delphi study to develop practical diagnostic guidelines for visual stress (pattern-related visual stress). *J. Optom.* 2017;10: 161-168 DOI: [10.1016/j.optom.2016.08.002](https://doi.org/10.1016/j.optom.2016.08.002). **DEM
You state “will be noted and inform …”. By “inform” do you mean “calculate”? Best to be explicit.**

**Author Response:** Thank you for this suggestion. The manuscript has been amended on line 369 to state that these metrics will be used to ‘calculate’ horizontal and vertical reading speeds.  **Statistical Analysis
I was unclear about several points here that could usefully be clarified for the reader:
(1) Are you going to report effect sizes and confidence intervals? (good practice)**

**Author Response:** Thank you for raising this point. On reflection and as discussed above, effect sizes and confidence intervals for change in reading speed on the Wilkins Rate of Reading Test with/without coloured lenses, will now be reported upon trial completion. This is discussed in lines 352 to 353.

**(2) Are you going to adjust for baseline measures?**

**Author Response:** Thank you for raising this query. We have updated the manuscript on lines 414 to 418 to state that we will consider baseline reading speed, the number of errors made in each reading test, number of days spectacle lenses worn, number of hours spectacle lenses worn and number of hours completing concentrated tasks with or without the use of coloured lenses, as covariates.

**(3) Are you going to test for carry over and period effects?
This can affect the analysis. For example, a 2x2 cross-over can be analysed with a t-test on the differences. ANOVA can be useful if you have different time points and want to look at the effect x time interaction (or other anticipated interactions). ANCOVA can be used when adjusting for baseline by including the baseline measures as covariates.**

**Author Response:** Thank you for your suggestion in how we can assess for carryover and period effects. As we believe that the washout period for this crossover study is ample (see the above response addressing washout times), we anticipate that carryover effects will be negligible and therefore won’t be included in our a priori statistical plan. If carryover effects are demonstrable upon study completion, post-hoc analysis could be conducted by a t-test, as suggested. This is addressed in lines 306 to 308 of the manuscript.

**In general, I think the proposed analysis could be explained more fully and more clearly.**

**Author Response:** Thank you for your feedback. We have added further information to the revised manuscript with more detailed statistical analysis and explanation of the statistical modelling we will conduct. **Data Management
Make it clear that it’s the (amended) Data Protection Act (2018). If the UK GDPR applies to your data also state you are complying with that.**

**Author Response:** We appreciate your feedback on this. We have amended the manuscript to include the appropriate Data Protection Act – see line 440. A comment on UK GDPR compliance has also been added to lines 449 to 451. **General
• Is this version 1 of the protocol. Can you report date and version number?**

**Author Response:** Thank you for your comment. This is version 2.0 of the protocol, effective as of 22/10/2022, as referenced in the change made in line 455.

**• I see you have a clinical trials manager but do you have a steering committee?**

**Author Response:** There is no formal steering committee, but the project is being managed by the chief investigator S.J.M. and study team co-investigator J.A.L., who periodically report on the progress of the study to the Centre for Optometry and Vision Science, and the School of Biomedical Sciences Human Intervention Studies (clinical trials) Unit. This has been detailed in lines 326 to 327.

**• Worth clarifying that this is a superiority trial**

**Author Response:** Thank you for identifying this. The manuscript has now been amended to include the phrase ‘superiority trial’ on lines 42 and 100.

**• Are you excluding those with a diagnosis of dyslexia? If not why not?**

**Author Response:** Thank you for this question. Those with formally diagnosed dyslexia and/or other Specific Learning Disability (SpLD), will not be excluded on the basis of these diagnoses. The rationale as to why these participants will not be excluded, is based on evidence within the literature, suggesting visual stress may occur as an isolated SpLD or may co-occur with another reading disorder. Presence of pattern glare and/or significant visuoperceptual reading symptoms will be used to identify symptomatic readers with assumed visual stress. This is addressed in lines 161 to 168 of the manuscript.

**• Are you matching groups for age, gender etc.?**

**Author Response:** Thank you for this comment. We will not be matching groups for age or gender. The studied cohort will be considered a homogenous cohort of undergraduate students. This is a cross-over trial so each participant acts as their own control, therefore there are no groups to match.

**• Do you have a Data Management Committee? If not state why.**

**Author Response:** Thank you for raising this point. Formally, there is not a data management committee. However, data collected within this trial will be handled by the chief investigator – S.J.M., as well as J.A.L. and D.H. applying University protocols for the management and securing of data.

**• What will you do with the data for those that don’t complete the trial?**

**Author Response:** Thank you for this comment. We will consider the percentage of missing data as a result of those who do not complete the trial. We have accounted for attrition in our sample size to mitigate the effect of drop out. If there are less than 5% missing data, this is considered negligible and only observed data will be analysed using complete case analysis. The limitations of the extent of the missing data will be discussed in any publications. Dependent on when the participant drops out of the study, observations can be analysed to the last observation recorded. We do not expect to need to use imputations where data are not available. This is detailed in lines 449 to 451 of the manuscript.

**• Do you plan to monitor adverse events? For example, what if (worst case) a participant wore the intervention while driving and had a RTA?**

**Author Response:** Thank you for raising concerns regarding adverse incidents. Participants will be advised not to use precision tinted spectacle lenses when driving at night-time, in line with manufacturer guidelines. In the unlikely event of adverse event related to spectacle wear whilst driving, incident(s) will be monitored and reported to the project’s chief investigator S.J.M.. Amendments have been made to the manuscript on line 460 to 463, explaining this.
