# Supplementary material for: Reading through the eyes of a university student: A double-masked randomised placebo-controlled cross-over protocol investigating coloured spectacle lens efficacy in adults with visual stress
Source: PLoS One. 2025 Jun 30;20(6):e0309625. doi: 10.1371/journal.pone.0309625 (PMC12208423; doi:10.1371/journal.pone.0309625)
Supplement: Attachment — Submitted filename: Response to Reviewers - February Submission.docx [file pone.0309625.s007.docx]

**RCT Protocol Paper – 20^th^ Jan 2025**

PONE-D-24-33918R2

Reading through the eyes of a university student: A double-masked randomised placebo-controlled cross-over protocol investigating coloured spectacle lens efficacy in adults with visual stress.

PLOS ONE

Dear Dr. Harkin,

Thank you for submitting your manuscript to PLOS ONE. After careful consideration, we feel that it has merit but does not fully meet PLOS ONE’s publication criteria as it currently stands. Therefore, we invite you to submit a revised version of the manuscript that addresses the points raised during the review process.

Please submit your revised manuscript by Mar 03 2025 11:59PM. If you will need more time than this to complete your revisions, please reply to this message or contact the journal office at [plosone@plos.org](mailto:plosone@plos.org). When you're ready to submit your revision, log on to https://www.editorialmanager.com/pone/ and select the 'Submissions Needing Revision' folder to locate your manuscript file.

Please include the following items when submitting your revised manuscript:

- A rebuttal letter that responds to each point raised by the academic editor and reviewer(s). You should upload this letter as a separate file labeled 'Response to Reviewers'.
- A marked-up copy of your manuscript that highlights changes made to the original version. You should upload this as a separate file labeled 'Revised Manuscript with Track Changes'.
- An unmarked version of your revised paper without tracked changes. You should upload this as a separate file labeled 'Manuscript'.

If you would like to make changes to your financial disclosure, please include your updated statement in your cover letter. Guidelines for resubmitting your figure files are available below the reviewer comments at the end of this letter.

If applicable, we recommend that you deposit your laboratory protocols in protocols.io to enhance the reproducibility of your results. Protocols.io assigns your protocol its own identifier (DOI) so that it can be cited independently in the future. For instructions see: <https://journals.plos.org/plosone/s/submission-guidelines#loc-laboratory-protocols>. Additionally, PLOS ONE offers an option for publishing peer-reviewed Lab Protocol articles, which describe protocols hosted on protocols.io. Read more information on sharing protocols at <https://plos.org/protocols?utm_medium=editorial-email&utm_source=authorletters&utm_campaign=protocols>.

We look forward to receiving your revised manuscript.

Kind regards,

Clara Martínez Pérez
Academic Editor
PLOS ONE


[Note: HTML markup is below. Please do not edit.]

Reviewers' comments:

Reviewer's Responses to Questions

**Comments to the Author**

1. Does the manuscript provide a valid rationale for the proposed study, with clearly identified and justified research questions?

The research question outlined is expected to address a valid academic problem or topic and contribute to the base of knowledge in the field.

Reviewer #1: Yes

Reviewer #3: Yes

2. Is the protocol technically sound and planned in a manner that will lead to a meaningful outcome and allow testing the stated hypotheses?

The manuscript should describe the methods in sufficient detail to prevent undisclosed flexibility in the experimental procedure or analysis pipeline, including sufficient outcome-neutral conditions (e.g. necessary controls, absence of floor or ceiling effects) to test the proposed hypotheses and a statistical power analysis where applicable. As there may be aspects of the methodology and analysis which can only be refined once the work is undertaken, authors should outline potential assumptions and explicitly describe what aspects of the proposed analyses, if any, are exploratory.

Reviewer #1: Yes

Reviewer #3: Partly

3. Is the methodology feasible and described in sufficient detail to allow the work to be replicable?


Descriptions of methods and materials in the protocol should be reported in sufficient detail for another researcher to reproduce all experiments and analyses. The protocol should describe the appropriate controls, sample size calculations, and replication needed to ensure that the data are robust and reproducible.

Reviewer #1: Yes

Reviewer #3: Yes

4. Have the authors described where all data underlying the findings will be made available when the study is complete?

The [PLOS Data policy](https://journals.plos.org/plosone/s/materials-and-software-sharing) requires authors to make all data underlying the findings described in their manuscript fully available without restriction, with rare exception, at the time of publication. The data should be provided as part of the manuscript or its supporting information, or deposited to a public repository. For example, in addition to summary statistics, the data points behind means, medians and variance measures should be available. If there are restrictions on publicly sharing data—e.g. participant privacy or use of data from a third party—those must be specified.

Reviewer #1: Yes

Reviewer #3: No

5. Is the manuscript presented in an intelligible fashion and written in standard English?

PLOS ONE does not copyedit accepted manuscripts, so the language in submitted articles must be clear, correct, and unambiguous. Any typographical or grammatical errors should be corrected at revision, so please note any specific errors here.

Reviewer #1: Yes

Reviewer #3: Yes

6. Review Comments to the Author

Please use the space provided to explain your answers to the questions above and, if applicable, provide comments about issues authors must address before this protocol can be accepted for publication. You may also include additional comments for the author, including concerns about research or publication ethics.

You may also provide optional suggestions and comments to authors that they might find helpful in planning their study.


(Please upload your review as an attachment if it exceeds 20,000 characters)

Reviewer #1: xxxxxxxxxxxxxxxxxxxxxxxxxxxxxxxxxxxxxxxxxxxxxxxxxxxxxxxxxxxxxxxxxxxxxxxxxxxxxxxxxxxxxxxxxxxxxxxxxxxxxxxxxxxxxxxxxxxxxxxxxxxxx

Reviewer #1:

We thank reviewer one for their time in reviewing the initial and revised version of this manuscript. We appreciate that no further comments have been raised by the reviewer. We wish to highlight their valuable contributions and we believe their insights have strengthened our manuscript.

Reviewer #3:

**Reviewer #3: A good quality study on this research question is very much needed. As the authors point out, many practitioners test and prescribe coloured lenses for visual stress, despite very weak evidence for this practice. I applaud the authors for planning this study. However I have a number of concerns as outlined below.**

**Author Response:** We thank reviewer #3 for their comprehensive and valuable comments, which we have used to strengthen our manuscript. We have addressed each of your concerns below individually. The manuscript has been marked up in red text where an amendment has been made in response to reviewer #3’s comments. Individual line number changes have been detailed in the comments below. **Page 4 line 84: theories (i) and (ii) seem to be mixed up here; reduction in hyperexcitation relates to theory (ii).
It is worth noting that while the hyperexcitation theory has been used as the basis for experiments on coloured filters in migraine, autism, ADHD and other conditions as well as visual stress, the theory is not well founded. FMRI experiments (Huang et al, 2011, referenced in this manuscript) have shown that in people with visual stress, coloured filters reduce activation slightly in one or two higher visual areas but not in V1, which is surprising and has not been well explained. In addition, another study (Kim et al 2015) has found increased activation in visual stress, so this picture is not at all clear and this should be acknowledged when discussing possible hyperexcitation.**

**Author response:** We appreciate this comprehensive suggestion exploring the aetiological theories of visual stress. We apologise for mislabelling theories (i) and (ii). These have now been amended in lines 81 to 86. We accept the reviewer’s comments as to how we can provide a more nuanced response in relation to the theory of cortical hyperexcitability. We have expanded this theory in lines 87 to 94.

**Page 5, line 96: there have been more than two systematic reviews that have concluded more RCTs are needed to establish whether coloured filters are beneficial in visual stress. The authors should review this again.**

Author response: Thank you for bringing this to our attention. We have now included further reviews and meta-analyses in the introduction in lines 99 to 102 of the manuscript.

**It is unclear how the control colour will be set – this should be specified more carefully.**

**Author response:** We thank the reviewer for their comment. Control tint will be set outside the ‘Limit of Therapeutic Effect’ – an area of proposed beneficial chromaticities mapped on the 1976CIELUV chromaticity diagram. We propose that this therapeutic limit will be individualised for each observer. As detailed in previous investigations, there are wide variations in the size of Just Noticeable Differences (JNDs) between observers when assessed on the intuitive colorimeter.

The control tint being placed outside the Limit of Therapeutic Effect is set to such a point that we propose will be inert for the participant. At the upper and lower boundary of the Limit of Therapeutic Effect, participants will consider if reading symptoms / visual discomfort experienced are ‘worse’ or ‘no different’ compared no colour presented. When participants confirm that the presented hue is ‘no different’ compared to no colour (i.e. not beneficial nor aversive), researchers propose that this be a candidate for the control tint. As the therapeutic area will have upper and lower boundaries, there will be two candidate control tints. The control tint chosen will be set outside the therapeutic limit closest to the optimal tint, to assist with participant masking – i.e. the apparent colour difference will be more alike between control and optimal tint. The manuscript has been adapted on lines 281 to 287, detailing these amendments. **Page 7 – near point of convergence ‘less than 10cm’ should probably be greater than 10cm – please check.**

**Author response:** Thank you for bringing this point to our attention. We were meaning that the poorer response would be an NPC distance greater than 10cm and we have amended this to ‘greater’ than 10cm as you suggest. This change is seen on line 166.

**The acuity criteria should apply monocularly and this should be specified.**

**Author response:** Exclusion criteria for monocular acuity (distance and near) have already been supplied in the manuscript – see lines 162 and 163.

**Reason for exclusion of photosensitive epilepsy should be explained.**

**Author response:** We have amended our exclusion criteria to explain the rationale behind diagnosis of photosensitive epilepsy as an exclusion criterion. This amendment is detailed in lines 171 to 173.

**I suggest that as well as taking into account deviation such as tropia and decompensating phoria, fixation disparity at distance and near should also be considered, and vergence reserves should be measured (it has been indicated that stereopsis, associated phoria and vergence reserves are key measures – see Evans et al 1995, 1996). I suggest fixation disparity may also be a useful addition criterion since it may explain some symptoms.**

**Author response:** We appreciate the comments made by the reviewer as to how we can enrich the binocular vision assessment of our participants prior to enrolment. Assessment of near associated phoria (by near Mallett unit) and measurement of positive, negative and vertical fusional reserves (measured by prism bar), will be conducted on all participants at baseline. Further binocular vision tests included at baseline include: calculation of the AC/A ratio (heterophoria method); accommodative facility (with +/-2.00D binocular flippers) and dynamic retinoscopy (with the Ulster-Cardiff cube). As the evidence base assessing the correlation between fusional reserves measures, associated phoria and visual stress is not definitive, we will not be using these tests to help determine exclusion criteria, but as further clinical tests conducted as part of a binocular vision work-up of each participant at baseline. An overview of the binocular vision work-up to be employed at baseline, has been detailed in lines 182 to 184 of the manuscript.

**Page 9 – it seems surprising that recruitment began two years ago. This being the case, has the study been underway for a while, and have the methods described here been used? If so, it may not be possible to apply the suggestions in this review. The purpose of a protocol is to declare methods before beginning data collection. Therefore if data collection has begun the purpose of this protocol seems unclear. Recruitment is to be completed by 20th December 24 (the date of writing this review) – this would be a long way into the students’ studies – if data collection is yet to begin, do they still want to participate, and if so the data collection would need to take place very soon if it is were to be completed while they are still at the University.**

**Author Response:** Owing to the relatively low assumed prevalence of visual stress in the adult population of ~15% and the large sample size required for this clinical trial, recurrent rounds of recruitment will be required in order to achieve sufficient participant recruitment numbers, if initial recruitment is sub-optimal. This amendment is seen on lines 223 to 225.

This investigation is seeking to ensure that our studied population is homogenous in nature, in order to draw meaningful conclusions from the outcomes of this trial. We wish to ensure our population is within a specific age range who all deal with a comparable level of reading demand – i.e. all participants are adult undergraduate students attending the same university in their first year of studies at time of initial trial recruitment.

We explore the reviewer’s reservations about the feasibility of publication in our next response. Yet, we can confirm that data collection has begun and participants have been motivated to take part. As the minimum length of time of an undergraduate course at Ulster University Coleraine is three years, all participants are still in attendance at university and therefore, trial completion is feasible within the remits of the timeframe of this study.

**Further to the above, on page 23 it is stated that 60 participants have been recruited and are enrolled in the trial. Again, this seems surprising prior to the publication of a protocol. The point of an RCT protocol is to set out the methodology before beginning the study. That way the methods are followed and not modified as the trial progresses; if the researchers decide to change their methods they record this change in the protocol (e.g. Tetzlaff et al 2012 Guidelines for randomized clinical trial protocol content: A systematic review). Publishing the protocol after the study has begun seems to miss the point that a clear protocol guides conduct of the trial. The authors should consider and explain the point of publishing a protocol at this stage.**

**Author Response:** When determining whether this protocol may be published in PLOSOne, we consulted the guidelines for publishing detailed by the journal: <https://journals.plos.org/plosone/s/submission-guidelines#loc-study-protocols>.

PLOSOne states that a clinical trial protocol must: (i) relate to a research study that has not yet generated results and (ii) be submitted before recruitment of participants or collection of data for the study is complete. We explore the reasons why our clinical protocol satisfy both criteria below:

(i) As this clinical trial is double-masked in nature and no statistical analysis shall take place until all participants have completed the trial, no results have been generated.
(ii) The clinical protocol has been submitted before recruitment of participants and collection of study data has been completed.

We would like to highlight that, to our knowledge, this will be the first clinical trial on precision tinted lenses to publish a clinical protocol in an academic journal, in advance of trial completion. We believe that this is of great importance for future academics to assist with replication of findings, if noteworthy in this investigation. Additionally, for context, we drafted and submitted this manuscript for publication some months ago, and have already been through a revision cycle.

**Page 10-11: The reading symptom score developed by the researchers seems to be key to visual stress diagnosis. As the authors point out, no clear system of diagnosis is available, but use of an unvalidated questionnaire will be a limitation of the study. If data collection has not yet begun, I suggest a pilot study should be conducted to test validity of this questionnaire.**

**Author response:** We agree with the reviewer, that currently no such validated visual stress questionnaire exists, and that the condition is diagnosed on an ad-hoc basis by eyecare practitioners. However, the items included in this reading symptom questionnaire were consolidated from a number of reading symptoms reported in the literature, proposed indicative of visual stress. We carried out further analyses on this questionnaire to assess its repeatability whilst screening large populations of students:

We conducted analyses into the test-retest reliability and internal consistency of the reading symptom questionnaire in order to confirm its validity. Cronbach’s alpha demonstrated high questionnaire internal consistency (α = 0.92). The questionnaire was retested on a subset of undergraduate students (n=115) after a period of 6-weeks. Retested participants were blinded to motivation for retesting as to avoid bias in responses. Furthermore, no participant with applicable reading symptoms was invited to partake in the clinical trial until after repeat testing had concluded, to reduce risk of biasing responses. Total summed symptom score two-way mixed average-measures intraclass correlation coefficient was deemed high (0.91). Bland-Altman analysis showed total summed questionnaire score bias was -1.99 (whereby a negative value indicated an increase on total summed score on repeat investigation). This suggests overall acceptable questionnaire test-retest reliability and internal consistency.

These findings have been briefly summarised in lines 199 to 206 of the manuscript.

References:

Bland JM, Altman DG. Statistical methods for assessing agreement between two methods of clinical measurement. *Lancet*. 1986;1: 307-310

**Questions on symptoms while reading include whether the letters appear jumbled and whether lines are skipped and the participant is invited to use the foreign language text to help them answer. It seems that most text in a foreign language would appear jumbled and difficult to follow smoothly. Have the authors considered using a language the participant understands? A reference is given to support the use of foreign language text but the rationale is not clear. In addition, will the same text be used each time symptoms are assessed? If so, there may be a slight learning effect as the participant becomes more familiar with it.**

**Author response:** To add some explanation of how the reading symptom questionnaire was structured, participants were asked to consider how their reading symptoms are when they read. As is detailed the supporting information in S2, the reading symptom questionnaire instructions do not explicitly ask participants to view the passage of foreign language text throughout the completion of the entire Likert scale section of the questionnaire (question one). The passage of crowded text is present to direct participants to when/if they struggle to answer a specific reading symptom question. The passage of crowded text acts as a visual aid for an uncomfortable ‘busy’ passage of text designed to evoke reading discomfort in symptomatic readers. This discomfort is probed specifically in question two of the questionnaire, where participants *will* be directed to view the passage of foreign text and document whether they do or do not experience any other reading symptoms when viewing the passage. Question two’s response was not considered as an inclusion criterion to the clinical trial, but will allow the researchers to assess the profile of symptomatic readers in post-hoc analysis as required.

To address the concerns related to potential learning effects of the foreign language passage; it is proposed that even with multiple exposures of written information through a unimodal input (i.e. solely visual input), foreign language vocabulary acquisition requires multiple exposures to new words in a meaningful context to acquire vocabulary (Bisson et al, 2014). We believe that the presentation of this unknown language to participants is not in a meaningful context and will reduce how likely participants are to acquire vocabulary, and therefore a learning effect be achieved. Participants were not directed to read the paragraph aloud nor was discussion surrounding the context of the foreign language passage facilitated, both of which are proposed to encourage vocabulary acquisition (Ulanoff & Pucci, 1999). Therefore, we believe that that any potential learning effects may be dampened by the fact that it will not be compulsory for participants to view the foreign language text upon repeat completions of the questionnaire.

References:

Bisson MJ, van Heuven WJB, Conklin K, Tunney, RJ. The Role of Repeated Exposure to Multimodal Input in Incidental Acquisition of Foreign Language Vocabulary. *Lang. Learning: J. Res. Lang. Studies.* 2014; 64(4): 855-877 DOI: [10.1111/lang.12085](http://dx.doi.org/10.1111/lang.12085)

Ulanoff SH, Pucci SL. Learning words from books: The effects of read aloud on second language vocabulary acquisition. *Bilingual Res. J.* 1999;23(4):409-422. DOI:

**Page 11: The determination of an arc of therapeutic effect is a new approach, to my knowledge, in which a range of similarly beneficial colours is identified. Again, this may be open to criticism unless validated by at least testing repeatability, and by establishing whether the control tint just outside of this arc is repeatedly not beneficial.**

**Author response:** We thank the reviewer for their comment but as they can appreciate there currently is no validated methodology to determine a placebo precision tint on the intuitive colorimeter, with previous investigations employing differing methodologies to determine sub-optimal tint.

We hypothesise that the separation between optimal and sub-optimal precision tints (i.e. the chromaticity difference – the Euclidean separation between u’ v’ coordinates on the 1976CIELUV chromaticity diagram), will not be uniform for all observers. Previous studies have applied a blanket chromatic separation between lenses to all participants (Huang et al, 2011, Coutts et al, 2012). We believe this methodology has limitations as the placebo tint is not individualised to the observer and could potentially be within the window of therapeutic effect or indeed aversive to the participant.

Therefore, using our clinical judgement we surmised that an individualised method which allows for personalised separation between tints for each participant, may be a more appropriate method to ensure that each participant receives a truly inert sub-optimal tint.

Encouragingly, when measuring mean chromatic separation between optimal and sub-optimal precision tint for those already enrolled in the clinical trial, we can confirm that average chromaticity difference remains at an acceptable separation of 0.062, in line with previous investigations (Wilkins et al, 1994, Wilkins et al, 2005, Aldrich et al, 2018). Therefore, we believe that we have adequately separated the precision tints for participants. We know the chromaticity difference for each individual and can use this to evaluate findings in post hoc analysis of reading performance, as required. Encouragingly, the mean chromatic separation seen in this study appears in line with previous investigations exploring ranges of separation whereby effect of precision tint is lost. Some previous investigations have been criticised for applying too small of a chromatic separation between lenses. Therefore, we believe that an appropriate level of separation is applied to our participants.

Furthermore, participants were graded at baseline on colorimeter reliability. Participants were classified as ‘good’ ‘moderate’ or ‘poor’ observers in relation to how easily they identified a precision tint on the colorimeter. These grading scales were derived by work of Aldrich et al (2018). Furthermore, if a participant was unable to determine a beneficial precision tint, they were excluded from the clinical trial. Amendments to the manuscript have been made to detail this in lines 307 to 312.

References:

Aldrich A, Lovell-Patel R, Allen PM, et al. The Repeatability of Colorimetry is Precise(ly) as Expected. *Neuro-Ophthalmology.* 2018;3: 1-6 DOI: 10.12691/novn-3-1-1

Wilkins AJ, Evans BJW, Brown JA, et al. Double-masked placebo-controlled trial of precision spectral filters in children who use coloured overlays. *Ophthalmic Physiol. Opt.* 1994;14: 365-370 DOI: <https://doi.org/10.1111/j.1475-1313.1994.tb00126.x>

Wilkins AJ, Shira N, Nimmo-Smith MI. How precise do precision tints have to be and how many are necessary? *Ophthalmic Physiol. Opt.* 2005;25: 269-276 DOI: [10.1111/j.1475-1313.2005.00279.x](https://doi.org/10.1111/j.1475-1313.2005.00279.x)  **Page 17: I suggest it may be helpful to audio record the WRRT test to allow researchers to check the result after the event. It is very difficult to accurately record mistakes, progress and completed time simultaneously, and live assessment of reading speed without a checking mechanism is likely to be flawed in my view.**

**Author Response:** We thank the reviewer for their comments. We conducted the Wilkins’ Rate of Reading Test in line with procedures employed in current clinical practice. The test has a proforma containing an identical version of test presented to the subject containing all words to be read. This allows the examiner to track and annotate any mistakes, repetitions or omissions made in real time while the participant reads the text aloud. This, coupled with a stopwatch with an alarm set to indicate when the minute has elapsed, enables the examiner to mark exactly how many words the participant has read. This proforma is used to calculate reading speed and errors made after testing. This method is in line with standard clinical practice, and a single examiner, experienced with conducting this test, will conduct all clinical trial visits (DH).

**Page 24: a reference is needed to support the statement that over 300 practices have an intuitive colorimeter, and other statements of fact in this section.**

**Author response:** Thank you for highlighting this. The manuscript has been altered on line 551 to now include a reference stating that there are over 300-practices in the UK with an intuitive colorimeter. We searched the Cerium website to determine how many practitioners within each UK postcode had an intuitive colorimeter. We then summed this number to get an estimate of the number of devices available in the entirety of the UK. We appreciate that there may be *more than* 300-intuitive colorimeters in the UK, but can confirm that at least 300 are currently listed by the device’s manufacturer.

7. PLOS authors have the option to publish the peer review history of their article ([what does this mean?](https://journals.plos.org/plosone/s/editorial-and-peer-review-process#loc-peer-review-history)). If published, this will include your full peer review and any attached files.


If you choose “no”, your identity will remain anonymous but your review may still be made public.


**Do you want your identity to be public for this peer review?** For information about this choice, including consent withdrawal, please see our [Privacy Policy](https://www.plos.org/privacy-policy).

Reviewer #1: No

Reviewer #3: **Yes:** Catherine Suttle

[NOTE: If reviewer comments were submitted as an attachment file, they will be attached to this email and accessible via the submission site. Please log into your account, locate the manuscript record, and check for the action link "View Attachments". If this link does not appear, there are no attachment files.]

While revising your submission, please upload your figure files to the Preflight Analysis and Conversion Engine (PACE) digital diagnostic tool, <https://pacev2.apexcovantage.com/>. PACE helps ensure that figures meet PLOS requirements. To use PACE, you must first register as a user. Registration is free. Then, login and navigate to the UPLOAD tab, where you will find detailed instructions on how to use the tool. If you encounter any issues or have any questions when using PACE, please email PLOS at [figures@plos.org](mailto:figures@plos.org). Please note that Supporting Information files do not need this step.
